# Vascular smooth muscle cell dysfunction in neurodegeneration

**DOI:** 10.3389/fnins.2022.1010164

**Published:** 2022-11-10

**Authors:** Genevieve Hayes, Joana Pinto, Sierra N. Sparks, Congxiyu Wang, Sana Suri, Daniel P. Bulte

**Affiliations:** ^1^Department of Engineering Science, Institute of Biomedical Engineering, University of Oxford, Oxford, United Kingdom; ^2^Department of Psychiatry, University of Oxford, Oxford, United Kingdom; ^3^Oxford Centre for Human Brain Activity, Wellcome Centre for Integrative Neuroimaging, University of Oxford, Oxford, United Kingdom

**Keywords:** vascular smooth muscle cells, neurodegeneration, cerebrovascular reactivity, cerebral blood flow, dementia, Alzheimer’s disease, MRI

## Abstract

Vascular smooth muscle cells (VSMCs) are the key moderators of cerebrovascular dynamics in response to the brain’s oxygen and nutrient demands. Crucially, VSMCs may provide a sensitive biomarker for neurodegenerative pathologies where vasculature is compromised. An increasing body of research suggests that VSMCs have remarkable plasticity and their pathophysiology may play a key role in the complex process of neurodegeneration. Furthermore, extrinsic risk factors, including environmental conditions and traumatic events can impact vascular function through changes in VSMC morphology. VSMC dysfunction can be characterised at the molecular level both preclinically, and clinically *ex vivo*. However the identification of VSMC dysfunction in living individuals is important to understand changes in vascular function at the onset and progression of neurological disorders such as dementia, Alzheimer’s disease, and Parkinson’s disease. A promising technique to identify changes in the state of cerebral smooth muscle is cerebrovascular reactivity (CVR) which reflects the intrinsic dynamic response of blood vessels in the brain to vasoactive stimuli in order to modulate regional cerebral blood flow (CBF). In this work, we review the role of VSMCs in the most common neurodegenerative disorders and identify physiological systems that may contribute to VSMC dysfunction. The evidence collected here identifies VSMC dysfunction as a strong candidate for novel therapeutics to combat the development and progression of neurodegeneration, and highlights the need for more research on the role of VSMCs and cerebrovascular dynamics in healthy and diseased states.

## Introduction

Cerebrovascular disease is among the leading disease-related causes of societal and economic burden in the developed world (Wang et al., 2016). The brain consumes 20% of the human body’s metabolic reserve, but it has a limited capability for energy storage. As a result, small changes in blood supply can lead to severe alterations in metabolic function, which can also lead to the secretion of neurotoxic and inflammatory factors, a reduction in cerebral blood flow (CBF), and hypoxia that might initiate and/or contribute to neuronal degradation (Zlokovic, 2011).

The aim of this review is to identify how vascular smooth muscle cells (VSMCs) participate in these vascular insults, and whether VSMC dysfunction may, in turn, contribute to neuronal degradation. Understanding the role of VSMC dysfunction in neurodegeneration is critical as it may inform on the underlying mechanisms behind cerebrovascular impairment that sometimes accompany and precede the onset of various neurological disorders.

In this review, we first summarise how VSMCs normally function to control cerebrovascular dynamics and blood flow, and key mechanisms by which VSMCs grow and communicate. Additionally, we discuss the best candidates for identifying the presence of cerebral VSMC dysfunction. We then present studies on VSMC phenotype, signalling pathways, and general contractility seen in a host of neurodegenerative disorders, presented in order of disorder prevalence. While it is not possible to encompass all neurodegenerative disorders, nor all the mechanisms at play, we review the role of VSMCs in Alzheimer’s disease (AD), Parkinson’s disease (PD), amyotrophic lateral sclerosis (ALS), multiple sclerosis (MS), Huntington’s disease (HD), cerebral small vessel disease (CSVD), post-stroke dementia (PSD), Down’s syndrome (DS), cerebral autosomal dominant arteriopathy with subcortical infarcts and leukoencephalopathy (CADASIL), Lewy body dementia (LBD), and Moyamoya disease (MMD). In assessing VSMC dysfunction in these disorders, the studies have only considered correlation not causation. Finally, we review how extrinsic influences can affect VSMC function and touch on a few potential therapeutic opportunities in the context of the evidence we have collected on VSMC dysfunction in neurodegeneration.

## Vascular smooth muscle cells control cerebrovascular dynamics

The vascular tree supplies the brain with blood flow and spans from the circle of Willis, which is known to be highly variable, down to the capillary network. The circle of Willis interconnects the anterior and posterior cerebral circulation network and gives rise to pairs of anterior, middle, and posterior cerebral arteries. Each artery divides into progressively smaller arteries and arterioles which run along the brain’s surface until they penetrate into the brain tissue (Ovsenik et al., 2021).

**FIGURE 1:**
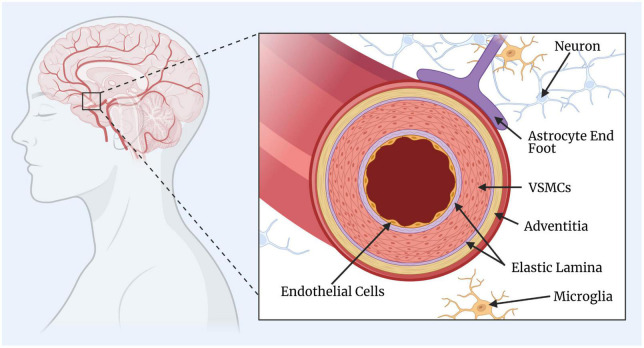
Simplified diagram of a cerebral artery. The key components of the cerebral artery include the endothelial cells, elastic lamina (one internal and one external), vascular smooth muscle cells (VSMCs), and the adventitia layer. The surrounding and supporting cells include the astrocytes, microglia, and neurons. This figure was created by GH rendering using BioRender.com.

Appropriate CBF is critical for brain function and survival. The regulation of CBF involves a coordinated interplay between different types of cells, including neurons, glia, and vascular cells. The neurons and glia generate signals which are translated into vascular changes by the collaboration of endothelial and mural cells. In particular, the mural cells, which include VSMCs and pericytes, surround the endothelial cell layer and regulate cerebrovascular resistance and blood flow to downstream capillary beds. Surrounding the smooth muscle is the adventitia layer that fuses to the basement membrane of astrocyte end feet, an important mediator of signals from neurons to blood vessels (MacVicar and Newman, 2015). In recent years, much attention has also been placed on the participation of glial cells, primarily astrocytes signalling pathways for cerebrovascular tone (Attwell et al., 2010; Filosa and Iddings, 2013). Together, these vascular and perivascular components make up the blood brain barrier (BBB), the selective interface between the blood and central nervous system (CNS). A simplified diagram of the anatomy of a cerebral artery is illustrated in Figure 1.

Vascular smooth muscle cells are a particular contractile cell type characterised by their expression of contractile proteins such as smooth muscle actin, myosin heavy chain, and myosin light chain (Poittevin et al., 2014). Through contraction and relaxation, VSMCs alter blood vessel diameter and enable the maintenance of appropriate cerebral blood pressure and flow. A cascade of events occurs in response to changing oxygen and nutrient needs, as chemical signals are converted by VSMCs into mechanical constriction or relaxation by inducing changes in calcium ion (Ca^2+^) concentrations, activating potassium channels, and altering the contractile state of the light chain of myosin. In response to mechanical forces, such as high circumferential stress, VSMCs undergo mechanical signalling to influence gene expression, regulate cellular function, and alter vascular tone, which occurs even faster than the chemical signal cascade and is known as mechanotransduction (Na et al., 2008; Liu and Lin, 2022). VSMCs are coupled by gap junctions which mediate the intramural propagation of vascular signals between cells (Iadecola, 2004). The renin-angiotensin system (RAS) is a key mediator of VSMC constriction and the Notch signalling pathway is essential in the coordination of VSMC migration and adhesion (Griendling et al., 1997; Sorrentino et al., 2022). Additionally, the free radical, nitric oxide (NO) is a powerful vasodilator, produced most readily through endothelial NO synthesis and is a vital component of the signalling cascade (Zhao et al., 2015). Notably, changes in these pathways may play a role in pathological states and are discussed in more detail below.

Some studies have reported that capillary pericyte contractility affects CBF as well, though at a much slower time-scale than VSMCs due to their lack of smooth muscle actin (Yemisci et al., 2009; Hall et al., 2014; Hartmann et al., 2021). While there are still unresolved controversies regarding pericyte contractility, it remains that in arteries and arterioles in the brain, VSMCs are likely to be the primary regulators of cerebrovascular dynamics and blood flow changes in response to the brain’s oxygen and nutrient demands (Hartmann et al., 2022).

### Vascular smooth muscle cell phenotypic plasticity

Vascular smooth muscle cells and endothelial cells exhibit a spectrum of genetically distinct phenotypes along the arteriovenous pathway, known as zonation (Vanlandewijck et al., 2018). Within arterial zones, VSMCs can also express different phenotypes that change their properties, assuring that each vessel can adapt to changes in the local conditions. VSMC phenotype modulation is affected by innate genetic programmes and environmental cues. Contractile and synthetic VSMCs represent the two ends of a spectrum of intermediate phenotypes that have clearly different morphologies. While many different phenotypes exist, the contractile and synthetic VSMCs are by far the most abundant and better researched than the others.

Contractile VSMCs are long, spindly, and contain lots of contractile filaments, while synthetic VSMCs are wider, with large extracellular matrices (Rensen et al., 2007). Contractile VSMCs are more abundant in healthy vessels than the synthetic VSMC phenotype. Furthermore, synthetic VSMCs tend to exhibit higher proliferative and migratory activity than contractile VSMCs, and increase local inflammation (Touyz et al., 2018). The VSMC phenotype and function can change in response to cell-signalling proteins, cell-to-cell contact, extracellular matrix interactions, injury stimuli, and mechanical forces. For example, immediately after an insult, VSMCs dedifferentiate, becoming more synthetic-like to promote the repair of the vessel. Then, once the injury is resolved, healthy VSMCs return to a non-proliferative, contractile phenotype (Davis-Dusenbery et al., 2011). In rare cases, VSMCs may also become hypercontractile due to excess contractile filaments which can increase the speed and amplitude of shortening (contraction) in response to stimulation and impair vasorelaxation (Ma et al., 1998).

**FIGURE 2:**
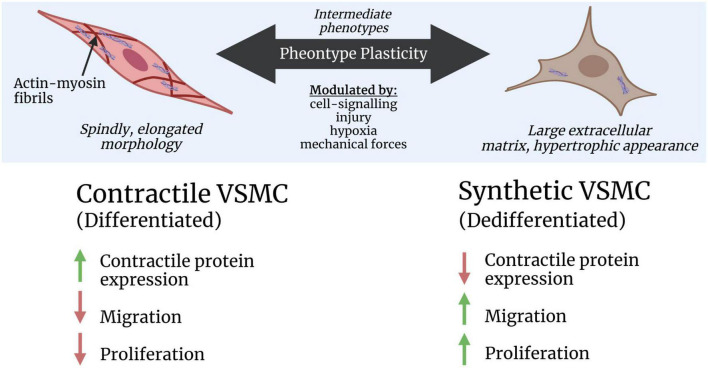
Simplified illustration of contractile and synthetic vascular smooth muscle cell (VSMC) phenotype plasticity. The key attributes for each phenotype are presented relative to each other, including their expression of contractile protein genes, migration, and proliferation. Higher levels are indicated by green, upward-facing arrows, and lower levels are indicated by red, downward-facing arrows. This figure was created by GH rendering using BioRender.com.

These transitions between the different phenotypes can be transmitted and regulated at multiple levels, including gene transcription, epigenetic modification, signal generation, and transduction (Frismantiene et al., 2018). It has been suggested that the switch to and from differentiated/contractile and dedifferentiated/synthetic phenotypes is a key element of disease progression and imbalanced VSMC plasticity might lead to progression of VSMC-driven vascular disorders (Frismantiene et al., 2018). An illustration of contractile and synthetic phenotypes is presented in Figure 2 along with their key attributes.

### Renin-angiotensin system in vascular smooth muscle cell function

The RAS is an important mediator of VSMC contractility (Griendling et al., 1997). When RAS is stimulated, the level of renin in the blood increases and promotes the production of angiotensin II (Ang II). Ang II is essential for the function and proliferation of healthy VSMCs but also plays a role in diseased states due to its growth-promoting effects on dysfunctional cells. The two major isoforms of the Ang II receptors, type-1 (AT1) and type-2 (AT2), appear to have opposing effects. Most of the known effects of Ang II are attributed to the AT1 receptors, which have a hypertensive effect, while AT2 receptors tend to produce hypotension (St. Paul et al., 2020).

The response of VSMCs to Ang II is multiphasic, first involving the mobilisation of Ca^2+^ and phospholipase C (Wynne et al., 2009). Phospholipase C mediates two distinct pathways which commonly result in VSMC contraction by activating several protein kinases, including myosin light chain kinase and Rho-kinase (Hilgers and Webb, 2005). The removal of Ca^2+^ from the cytosol and the increase in myosin phosphatase initiate VSMC relaxation.

The prolonged effect of Ang II binded to AT1 receptors is the activation of NADH/NADPH oxidase which stimulates the intracellular formation of reactive oxygen species (ROS) such as superoxide anions (Griendling et al., 1997). While their precise mechanisms remain unexplored, ROS appear to play an important role in VSMC proliferation, DNA synthesis, and apoptosis (Rao and Berk, 1992; Tsai et al., 1996).

### Notch signalling in vascular smooth muscle cells

Notch receptors in VSMCs appear to promote the phenotypic transition toward a contractile, differentiated state and promote VSMC survival (Baeten et al., 2015; Baeten and Lilly, 2017). The Notch signalling pathway is one the prominent communication routes between vascular cells and has been found to be upregulated in VSMCs after vessel injury, particularly in VSMC regions close to endothelial cells in the regenerating endothelium (Lindner et al., 2001). Notch signalling is reiteratively used in VSMCs to positively regulate differentiation, arterial specification, and maturation. The connection between notch signalling and vasculature was first recognised when dominant mutations in the Notch3 receptor were found to be responsible for CADASIL (Joutel et al., 1996).

## Characterising vascular smooth muscle cell dysfunction *in vivo*

A pure representation of VSMC dysfunction can be identified at the molecular level both preclinically, and clinically *ex vivo*, however *in vivo* methods are necessary for the identification of VSMC dysfunction in patients when interventions may still be possible. Characterising VSMC dysfunction can be difficult *in vivo* due to the high number of physiological and environmental parameters affecting *in vivo* measurements. Here, we will discuss the most promising parameters and techniques for identifying the presence of cerebral VSMC dysfunction.

### Cerebral blood flow and cerebrovascular reactivity

Vascular smooth muscle cells directly participate in the regulation of CBF and due to their contractile properties, they largely mediate vascular contraction and relaxation (Hill et al., 2015). Decreased baseline CBF correlates with the severity of cognitive symptoms of dementia, showing the potential contribution of CBF alteration to cognitive decline (Bracko et al., 2020).

Cerebrovascular reactivity (CVR) is an indicator of the intrinsic ability of brain vessels to contract or dilate in response to vasoactive stimuli and therefore characterises the brain’s ability to support neuronal function under stress. Changes in vessel calibre occur in response to a stimulus, such as alterations in the brain’s oxygen/nutrient requirements, or changes in the partial pressure of carbon dioxide (CO_2_). As a result, CVR might provide additional and more sensitive information on brain health compared with baseline CBF. For example, in a comparison of magnetic resonance imaging (MRI)-derived CVR and CBF using arterial spin labelling (ASL) in healthy adults and adults with mild cognitive impairment (MCI), whole-brain CVR in both grey and white matter was positively associated with cognitive performance in response to hypercapnia, even in the absence of a statistical relationship between baseline CBF and cognitive performance (Kim et al., 2021).

Furthermore, because the dilation of cerebral blood vessels, known as vasodilation, is induced during CVR measurements, CVR may also be more representative of VSMC function and morphology than baseline CBF.

### Imaging cerebral blood flow and cerebrovascular reactivity

Whole brain CBF and CVR mapping can be acquired using positron emission tomography (PET) and single-photon emission computed tomography (SPECT), however, they require the administration of radiotracers, limiting their ease of use and appropriateness in many studies. Non-invasively, CBF and CVR can be measured using MRI methodologies such as ASL and blood-oxygen-level-dependent (BOLD) imaging (Pinto et al., 2021). ASL allows direct and quantitative measurement of CBF, while the BOLD signal results from a combination of several physiological parameters (CBF, cerebral blood volume, and cerebral metabolic rate of oxygen). When using a challenge that increases the arterial blood partial pressure of CO_2_, it is assumed that the BOLD signal changes predominantly result from changes in CBF. It should also be noted that the BOLD signal has a better signal-to-noise ratio, higher temporal resolution, and is more widely available in conventional MRI scanners than ASL (Catchlove et al., 2018). Nevertheless, CVR and CBF quantification obtained using MRI techniques have been shown to have similar accuracy and precision to ^15^O H_2_O PET, which is considered to be the gold-standard measurement of CBF (Heijtel et al., 2014; Hauser et al., 2019). Hauser et al. (2019) found that CO_2_-triggerent BOLD MRI correlates strongly with ^15^O H_2_O PET acetazolamide challenge. Heijtel et al. (2014) compared ASL MRI and PET in both baseline and CO_2_-induced hypercapnia, and found intra- and inter-session measurements to be highly comparable with similar precision.

Transcranial doppler (TCD) ultrasound, near infrared spectroscopy (NIRS) can also be used as non-invasive and indirect measures of CBF regulation and CVR (Ainslie and Duffin, 2009; Amyot et al., 2022). TCD measures blood velocities within an artery, often the middle cerebral artery, as a proxy for CBF. Although it is inexpensive, portable, and relatively easy to use, TCD has low spatial resolution and cannot be used for regional analysis. NIRS provides a real-time assessment of fluctuations in cerebral blood haemoglobin differences in large blood vessels, which is highly correlated with CBF (Tsuji et al., 1998). It should be noted that localised pathologies may not manifest as global changes in cerebrovascular dynamics, and therefore CVR measured using TCD and NIRS might not show changes, while regional CVR measured using PET, SPECT, or MRI may. Other imaging techniques exist for the quantification of CBF in preclinical models including two-photon fluorescence microscopy and laser Doppler flowmetry which can both image the motion of red blood cells in microvasculature and at the subcellular level in the cortex *in vivo* (Dirnagl et al., 1989; Kleinfeld et al., 1998).

### Methods for inducing hypercapnia and hypocapnia

The most common vasoactive challenge used for CVR assessment involves inducting hypercapnia and/or hypocapnia. In hypercapnia, the arterial blood partial pressure of CO_2_ is increased leading to vasodilation and increased CBF. This is achieved by the direct effect of CO_2_ and decreased extracellular pH on VSMCs to open their potassium channels, resulting in hyperpolarization (Brayden, 1996). This decreases the activity of voltage-dependent Ca^2+^ channels, thus decreasing intracellular Ca^2+^ and leading to VSMC relaxation and vasodilation (Pfefferkorn et al., 2001). Hypocapnia represents the inverse, whereby the arterial blood partial pressure of CO_2_ is decreased leading to vasoconstriction. Cerebral vasodilation and vasoconstriction requires the orchestration of the whole neurovascular unit including endothelial cells, astrocytes, parenchymal neurons, and perivascular nerves, in addition to VSMCs (Lavi et al., 2006; Iadecola, 2013; Haidey et al., 2021). Therefore, VSMC dysfunction cannot be completely isolated under hypocapnic and hypercapnic conditions, however their functionality may still be well characterised under these conditions as they change and maintain vascular tone (Brian, 1998; Cipolla, 2009).

One well-known method of inducing hypercapnia in subjects is the inhalation of air with increased CO_2_ content. Different techniques have been developed in order to more precisely control CO_2_ concentrations, including those based on fixed inspired CO_2_ (Liu et al., 2017). Some more complex respiratory gas manipulation techniques allow for the precise targeting and maintenance of end-tidal carbon dioxide (PETCO_2_), a non-invasive surrogate for the corresponding arterial gas concentration (Spano et al., 2013). Since room air has very low concentrations of CO_2_, hypocapnia is usually achieved through hyperventilation to increase CO_2_ removal from the blood. PETCO_2_ is derived from air expelled from the lungs during hypocapnia or hypercapnia. PETCO_2_ and CVR exhibit a sigmoidal relationship which is best modelled using progressively applied levels of hypo-/hypercapnia (Bhogal et al., 2014).

Hypercapnic vasodilation can also be induced with exogenous chemicals such as acetazolamide, which can be done in a single dose with a high reproducibility. In most cases, the administration of CO_2_ is still preferred over intravenous acetazolamide because the final serum concentration for a given dose of acetazolamide and the cerebrovascular responses to that serum level can vary considerably between subjects. These variations are reduced at higher concentrations of acetazolamide, but higher concentrations also increase the severity and frequency of side-effects and subject discomfort (Grossmann and Koeberle, 2000).

Strategies based on breathing tasks have also been used, such as breath holding or paced deep breathing that can readily induce changes in arterial pressure of blood gases and consequently drive hypercapnia (vasodilation) and hypocapnia (vasoconstriction), respectively. Breathing tasks are often simple and inexpensive to implement, therefore they may offer an accessible option for CVR mapping. However due to the many factors that affect arterial blood CO_2_ other than the breathing rate, subject variability must be considered to make this a reliable stimulus to identify cerebrovascular pathology (Fierstra et al., 2013).

## Vascular smooth muscle cell dysfunction and neurodegenerative disorders

Many neurological disorders share a common pathological triad of vascular damage, neuronal degeneration, and neuroinflammation. This vasculo-neuronal-inflammatory triad was termed by Zlokovic and colleagues, and involved not just neurons, but brain endothelium, VSMCs, pericytes, astrocytes, and activated microglia (Zlokovic, 2011). Specifically, many brain disorders share VSMC dysfunction as a mechanism for the progression of neurodegenerative pathology. Although there may be overlap between many of these pathologies, here we discuss and summarise existing research on the role of VSMCs in prevalent neurodegenerative disorders.

### Alzheimer’s disease

Alzheimer’s disease is characterised by progressively impaired memory, cognition, and behavioural changes, and it is the most common cause of dementia. Of the three polymorphic alleles of the human apolipoprotein E (APOE) gene, ε2, ε3, and ε4, the presence of APOE ε4 has been identified as the most prominent genetic risk factor for late-onset AD (Bertram et al., 2007). Notably, increasing evidence indicates that APOE genotypes differentially modulate cerebrovascular function, and that APOE ε4 induces detrimental cerebrovascular effects including reduced CBF, increased BBB leakiness, and disrupted transport of nutrients and toxins (Alonzo et al., 1998; Zerbi et al., 2014; Halliday et al., 2016).

Decreased VSMC density was identified in the blood vessels of the arachnoid, grey matter, and white matter of patients with AD compared with age-matched, healthy controls (Ervin et al., 2004). Patients with AD expressing the APOE ε4 gene also have less smooth muscle actin immunoreactivity, associated with more severe AD, than patients with AD expressing APOE ε3 (Ervin et al., 2004). Furthermore, APOE ε4 is associated with impaired intramural periarterial drainage (IPAD) in mice (Hawkes et al., 2012).

Studies have reported that in patients with AD and mouse models of AD, the overexpression of molecules that control the rate of cell transcript in cerebral VSMCs generates a hypercontractile VSMC phenotype with reduced clearance receptors for the notorious amyloid-beta (Aβ) peptides (Chow et al., 2007; Bell et al., 2009). Autoimmune response mechanisms may also be involved in the degeneration of VSMCs during the development of AD. For example, an increase in anti-smooth muscle antibodies was found to be strongly correlated with brain atrophy in patients with early AD (Giulia et al., 2015).

Furthermore, specific genes have been associated with changes in VSMC in AD. An upregulation of synthetic-VSMC genes has been found in patients with AD, while the expression of VSMC contractile-genes is significantly reduced in AD cerebral pathology compared to healthy brains (Owens et al., 2004; Mack, 2011; Frismantiene et al., 2018). Notably, changes in the gene encoding for the sarco-endoplasmic reticulum calcium ATPase isoform 2 (SERCA2), a gatekeeper of normal VSMC function, have been associated with AD (Britzolaki et al., 2020). The overexpression of SERCA2b was shown to lead to elevated Aβ production, while the knockdown of SERCA2b caused a drastic decline in Aβ (Green et al., 2008). Gallego et al. (2018) further support the importance of maintaining SERCA activity and show that dysfunctional SERCA can have damaging effects on VSMC Ca^2+^ homeostasis causing AD-associated neuronal cell death.

Growing evidence shows that cerebrovascular contractility may be impaired in AD as well as in MCI, a stage of cognitive decline more severe than what is expected through normal ageing, but less serious than the decline seen in AD. People with MCI are significantly more likely to progress to AD and dementia than healthy adults and are therefore a group of interest for better understanding the development and progression of AD (Flanagan et al., 2016). Several studies have shown that neurocognitive decline in MCI and AD is paralleled by reduced CVR and deficits in vascular contractility, identified using hypercapnic challenge in CO_2_ BOLD MRI, TCD, and PET imaging (Vicenzini et al., 2007; Cantin et al., 2011; Yezhuvath et al., 2012; Richiardi et al., 2015). A recent investigation using ASL MRI showed that impaired CVR in grey matter regions and the whole brain white matter may be an early imaging biomarker revealing the relationship between cerebrovascular function and cognitive decline (Kim et al., 2021). However, other studies have shown that baseline CBF and global CVR are preserved in AD (Jagust et al., 1997; Rodell et al., 2012; Glodzik et al., 2013). Rodell et al. (2012) found that after factoring out the effects of CO_2_ on blood flow, people with AD no longer showed significant inter-subject global CBF variability, though reduced water and oxygen clearance was reduced in specific regions of the brain. While clinical studies are not yet conclusive, there is a substantial body of research that highlights that CVR may be a prospective means to detect early vascular dysfunction in subjects at risk (Glodzik et al., 2011). However, it is also clear that the way in which CVR is measured must be standardised to make it clinically useful.

A common pathological feature associated with AD is cerebral amyloid angiopathy (CAA). CAA results from the build-up of Aβ in the basement membrane of VSMCs in cerebral arteries and the basement membrane of cortical capillaries. Studies have demonstrated that VSMCs can undergo dramatic phenotypic transitions in CAA-like neuroinflammatory conditions, adopting synthetic and proliferative phenotypes (Aguilar-Pineda et al., 2021b). Additionally, these phenotypic transitions often occur in conjunction with tau protein hyperphosphorylation and accumulation in the brain (Aguilar-Pineda et al., 2021b).

Aldea et al. (2019) have studied the impact of vasomotion, a rhythmic relaxations and contractions of cerebral vasculature of ∼0.1 Hz, on the IPAD mechanism in CAA, and propose that the failure of the vasomotion-driven IPAD mechanisms are responsible for the progression of CAA (Rayshubskiy et al., 2014). Their mathematical models showed that the contractile VSMCs of cerebral arteries act as the drivers of IPAD in the brain by inducing basement membrane deformation in the direction of the vasomotion, contributing to the drainage of fluid and soluble metabolites from the brain. It has been suggested that the force of contractile VSMCs in arteries and arterioles contribute to drainage along the IPAD pathway all the way down to the capillary level, and that impaired VSMCs could result in the accumulation of Aβ in the walls of macro- and microvasculature, contributing to CAA pathology (Aldea et al., 2019). In CAA, Aβ peptides may also contribute to the transition of VSMCs from contractile to synthetic phenotypes. One study showed that VSMCs exposed to Aβ1-40-peptide, the main amyloid peptide found to accumulate in the vessel wall in sporadic forms of CAA before the addition of proinflammatory cytokines, was greatly intensified compared to those only exposed to the cytokines (Vromman et al., 2013). Additionally, changes in transcription factors were suggested as a possible path leading to VSMC phenotypic changes and vascular dysfunction in CAA.

Further research into VSMC function and signalling in AD and CAA pathologies is required to better understand the mechanisms underlying vascular and neuronal degradation. However, the cumulation of these findings support that VSMC dysfunction may be a potential biomarker for characterisation or treatment during the development and progression of disease.

### Parkinson’s disease and lewy body dementia

Parkinson’s disease is characterised by the presence of intracellular Lewy bodies containing alpha-synuclein (aSyn) fibrils, which are thought to lead to the cell death of dopaminergic neurons in the substantia nigra, affecting motor control, tremors, and gait (Yasuda and Mochizuki, 2010). The mechanism by which aSyn causes neurodegeneration is unclear, however these fibrils are linked to mural cell function because VSMCs and pericytes appear to be able to take up, secrete, and transfer aSyn (Tamo et al., 2003; Fields et al., 2019). A loss in BBB integrity has been observed post-mortem and it has been suggested that aSyn may be responsible for the breakdown of mural and endothelial cells in the BBB (Dohgu et al., 2019).

The specific role of VSMC dysfunction is not yet clear in PD pathologies. One study showed that the global response of CBF and CVR did not differ significantly between patients with PD and healthy controls, though a correlation was identified between increased CBF in posterior regions of the brain and severity of cognitive impairment in the PD group (Al-Bachari et al., 2014). Another study also did not find any significant difference between the whole-brain CBF and CVR measurements between patients with PD and health controls (Pelizzari et al., 2019). However, similar to Al-Bachari et al. (2014), Pelizzari’s group found that regional increases in CBF correlated with the severity of PD motor symptoms, most significantly in the postcentral gyrus. Furthermore, significant negative correlations were found between CVR and PD severity, corrected for age, in specific regions of the brain, such as the corpus striatum, which is involved in the generation and inhibition of movement.

Genetics also play a role in PD pathogenesis, such as protein deglycase Dj-1 and NDUFV2 (Rui et al., 2018; Lin et al., 2021). Dj-1 is a gene accountable for the autosomal recessive early onset form of PD and is multifunctional (Bandopadhyay et al., 2004; Clements et al., 2006). Mutations in Dj-1 result in neurodegeneration, leading to an early onset familial form of PD (Bonifati et al., 2003). Dj-1 also responds to oxidative stress in VSMCs, inhibits hyperplasia, and maintains vasorelaxation by participating in endothelial NO synthesis, though the effects of Dj-1 in VSMC phenotype switching remain unclear (Won et al., 2013). The NDUFV2 gene, also involved in AD pathogenesis, contributes to VSMC communication and may play a role in phenotypic switching to synthetic, pro-inflammatory phenotypes (Gan et al., 2017). Furthermore, like with AD, changes in the gene encoding for SERCA2 have recently been associated with aSyn and PD pathology. The investigators proposed that abnormal accumulation of aSyn could lead to intracellular Ca^2+^ dyshomeostasis due to the downregulation of SERCA2 activity in preclinical models (Tuon et al., 2012).

Lewy body dementia is a type of dementia in which the same aSyn Lewy bodies found in PD clump in the cytoplasm of neurons, which disrupts the production of dopamine and causes cognitive decline often as well as motor symptoms such as muscle weakness and rigidity. The role of VSMCs in LBD has been very minimally studied and is largely based on evidence of changes to general cerebrovascular function. Reductions in CBF and microvessel density associated with deficient vascular endothelial growth factor have been described in the occipital cortex of LBD patients compared with controls, though it was unclear whether this was secondary to the accumulation of aSyn (Miners et al., 2014). The contribution of hypoxia and hemodynamic changes has been hypothesised based on the high levels of cortical microinfarcts in LBD which is also found in CAA and vascular dementia (Reuck et al., 2014). LBD patients appear to experience a significant inflammatory response and vascular abnormalities, which exacerbate Lewy body-induced neuropathology, though specific research on VSMCs in LBD has not yet been explored (Raz et al., 2015). Changes in CVR and CBF mechanisms have yet to be investigated in patients with LBD.

### Amyotrophic lateral sclerosis

Amyotrophic lateral sclerosis is a progressive neurodegenerative disease of the motor system characterised by focal to generalised weakness leading to paralysis and respiratory failure (Peters and Brown, 2015).

Patients with ALS can exhibit more than a 50% reduction in pericyte numbers in the spinal cord and a significant increase in platelet-derived growth factor (PDGF) expression, a potent protein, which enhances the rate of cell division and is involved in VSMC recruitment, differentiation, and homeostasis (Winkler et al., 2013). PDGF also induces VSMC phenotypic switching from a contractile to proliferative and proinflammatory state, and it has been suggested that this plays an important role in atherosclerosis (Krettek et al., 1997; Zhao et al., 2011).

There have been very few studies that have investigated the role of VSMC dysfunction in ALS pathology, however it has been speculated that VSMCs could be involved in ALS due to upregulation of Ang II in the CNS (Sorrentino et al., 2022). This is accompanied by a significant reduction in Ang II found in cerebrospinal fluid (CSF) from patients with ALS, which has been associated with disease severity and progression rates (Kawajiri et al., 2009). This indicates that changes to RAS and Ang II levels may induce VSMC dysfunction and ALS pathology, but more research is required to better understand the mechanisms and integrity of VSMCs and cerebrovascular dynamics in ALS.

### Multiple sclerosis

Multiple sclerosis is a complex inflammatory autoimmune disease, largely affecting the white matter of the CNS characterised by demyelination, axonal loss, and the formation of focal and diffuse lesions (Hernandez et al., 2014, p. 52).

Compared with other neurodegenerative diseases, the role of VSMCs in MS progression is not very well understood, though it’s clear that Ang II plays a role in the pathogenesis of MS. Enhanced levels of Ang II in the CNS are culprits of vascular disease and inflammation, and are also seen in MS along with decreased CSF Ang II and increased serum angiotensin-converting enzyme (Guzik et al., 2007; Matsushita et al., 2010). Increased CNS Ang II was also found in a mouse model of MS accompanied by an upregulation of AT1 receptors in MS brain lesions (Platten et al., 2009). In VSMCs, upregulated AT1 can promote hypertensive effects and increase ROS activity.

A significant decrease in grey matter CVR has been shown in patients with MS compared to healthy controls (Marshall et al., 2014). The same study found that impaired CVR was significantly correlated with increased lesion volume and grey matter atrophy, suggesting that impaired vasodilation may be an underlying cause of neurodegeneration in MS. More research is needed to properly understand the role and mechanisms of VSMC dysfunction and altered vascular dynamics in MS.

### Huntington’s disease

Huntington’s disease is an autosomal-dominant neurodegenerative disorder affecting the CNS, which can lead to involuntary choreiform movements, personality changes, and dementia (Parsons and Raymond, 2015).

The role of VSMCs in HD is not yet conclusive and is largely based on preclinical studies. Models using mice expressing the huntingtin gene indicate that vascular dysfunction might develop earlier in life in the HD mice than in wild-type mice (Rahman et al., 2013). The HD mice presented with reduced contractility in arteries of varying vascular beds, attributed to altered Ca^2+^ fluctuations in the arterial VSMCs. However, in a different HD mouse model, the response to potassium chloride was tested as a global measure of vascular smooth muscle structure, mass, and function, and no difference was found compared to wild-type mice (Kane et al., 2016).

Studies have also investigated the role of myosin phosphatase target subunit 1 and Rho kinase in HD (Narayanan et al., 2016). Myosin phosphatase target subunit 1 is essential for VSMC relaxation through the regulation of myosin light chain phosphatase, and Rho kinase contributes to Ca^2+^ sensitization in VSMC contraction. Both the myosin phosphatase target subunit 1 and Rho kinase were significantly increased in patients with HD and were found to lead to increased apoptotic cell death (Narayanan et al., 2016). More research is required to better understand the implications of VSMC dysfunction and their molecular pathways in HD.

### Down’s syndrome

Down’s syndrome is caused by complete or partial trisomy of chromosome 21, frequently causing intellectual disability and reduced lifespan (Parra et al., 2017). People with DS are at a high risk of developing AD at an early age, but are much less likely to develop cardiovascular disease than those without, despite DS being associated with premature ageing, the early onset of AD, and a higher prevalence of risk factors for cardiovascular disease such as obesity, dyslipidemia, and sedentarism (Murdoch et al., 1977; Sobey et al., 2015; Parra et al., 2017).

Structural MRI studies have shown notable increases in cerebrovascular pathology in people with DS, including lobar microbleeds, infarcts, and white matter hyperintensities (WMHs), not strongly associated with Aβ pathology (DiProspero et al., 2022). CBF is significantly lower among adults with DS with probable AD compared with controls and participants with DS without dementia when measured using pulsed ASL (Thalman et al., 2020). The implications of VSMCs on these changes have not yet been explored.

Two genes are found on chromosome 21 in DS that appear to play a role in vascular health, neural development, and the onset of AD: DYRK1A and RCAN1 (Roy-Vallejo et al., 2020). DYRK1A has recently been shown to decrease neprilysin enzyme levels in subjects with DS (Kawakubo et al., 2017). Neprilysin degrades natriuretic peptides and vasodilators, which are negatively correlated with VSMC migration and proliferation, and the inhibition of neprilysin is related to the accumulation of Aβ peptides and the development of AD in adults with DS (Garg and Hassid, 1989; Miners et al., 2011; Karoor et al., 2013). Interestingly, it has the opposite effect in cardiovascular disease where its inhibition has been shown as an effective treatment for heart failure and hypertension (von Lueder et al., 2014; Vodovar et al., 2015). The effect of RCAN1 on VSMC phenotype is unclear, though it appears to play a role in VSMC migration. The genetic ablation of RCAN1 in mice led to decreased VSMC migration and resistance to Ang II induced aneurysm and restenosis (Esteban et al., 2011). In contrast, endogenous RCAN1 knockout was found to increase migration in cancer cells *in vitro*, whereas the expression of exogenous RCAN1 reduced migration (Espinosa et al., 2009). RCAN1 thus appears to have different effects on cell migration depending on the setting.

## Vascular smooth muscle cell dysfunction and cerebrovascular pathology

Here we review the existing research on the role of VSMCs in pathologies that are well known to present a clear vascular component: CSVD, CADASIL, stroke, PSD, and MMD.

### Cerebral small vessel disease and cerebral autosomal dominant arteriopathy with subcortical infarcts and leukoencephalopathy

Cerebral small vessel disease is composed of several diseases affecting the small arteries, arterioles, capillaries, venules, and small veins, which may include genetic, idiopathic, infectious, and immune-related pathologies (Cannistraro et al., 2019). While the manifestation and progression of CSVD is broad reaching, VSMC migration and hypertrophy due to the upregulation of Ang II and leading to increased ROS may be a common link to vascular remodelling and dysfunction (Virdis et al., 2004). In preclinical and *in vitro* studies of Ang II-induced CSVD, ROS have been shown to alter blood pressure, vascular structure, and collagen deposition (Griendling et al., 1994; Virdis et al., 2004).

Beyond investigating the role of Ang II and ROS in VSMC function in CSVD, much of the research on VSMCs in CSVD has been based on CVR and CBF studies. It has recently been demonstrated that patients with advanced hypertensive CSVD have impaired CVR in the basal ganglia, temporal lobe, and frontal lobe, determined using ASL MRI with an intravenous vasoactive stimulus (Lee et al., 2021). The same study found that the reduced vasoconstriction was significantly associated with conventional MRI markers of CSVD, including cerebral microbleeds, WMHs, and deep lacunar infarcts. Furthermore, lower CVR determined using BOLD MRI under hypercapnic challenge was found to be indicative of WMHs and perivascular spaces associated with CSVD when controlling for patient characteristics (Blair et al., 2020). Another BOLD MRI study showed that areas of reduced CVR precede the progression of normal-appearing white matter to WMHs suggesting that impairment of vascular contractility may contribute to the pathogenesis and progression of CSVD (Sam et al., 2016). A systematic review of CSVD and ischaemia associations showed that baseline CBF was also negatively related to WMH severity linked to CSVD, and Shi et al. (2016) noted that investigating CVR in patients with CSVD may provide new insights.

Cerebral autosomal dominant arteriopathy with subcortical infarcts and leukoencephalopathy is a rare, hereditary CSVD characterised by VSMC degradation and the accumulation of Notch3 receptors on the VSMCs of small and middle-sized arteries (Joutel et al., 2000, 1997). A common pathological feature of CADASIL is the dramatic reduction of VSMCs in cerebral arterioles walls, which may be attributed to irregular mitochondrial proliferation and function in CADASIL VSMCs (Viitanen et al., 2013).

In a study comparing proteomic expression of cultured CADASIL and control VSMCs isolated from human umbilical cord, it was found that CADASIL is likely caused by misfolded Notch3 molecules (Ihalainen et al., 2007). This impairs the Notch3 signalling cascade, increases ROS, inhibits cell proliferation, and degrades VSMCs. Ihalainen et al. (2007) found that this also induces the overproduction of collagen type I, leading to fibrosis, vascular stenosis, and ischaemic lesions as seen in patients with CADASIL. Similarly, cultured CADASIL VSMCs have been shown to have a lower proliferation rate compared to control VSMCs and also showed an increased gene expression of transforming growth factor-beta that, when neutralised, corrected the proliferation rate (Panahi et al., 2018).

The CVR and mean CBF in the middle cerebral artery, measured using TCD and MRI, have been shown to be reduced in CADASIL compared with controls, attributed to VSMC dysfunction (Pfefferkorn et al., 2001; Liem et al., 2009; Moreton et al., 2018; Atwi et al., 2019). Pfefferkorn et al. (2001) also found reduced CO_2_ reactivity in patients with CADASIL before they became disabled, suggesting that CVR plays an early role in the evolution of the disease. In an MRI analysis using an intravenous acetazolamide vasoconstrictive challenge, CVR was significantly reduced in CADASIL and was identified as an important determinant of development of WMHs (Liem et al., 2009). Furthermore, an overall decrease in relative CBF and cerebral blood volume, before and after an acetazolamide challenge, were detected in patients with CADASIL when measured using MRI (Chabriat et al., 2000).

There have also been studies that found that CVR was not impaired in patients with CADASIL when measuring large arterial flow using MRI and TCD (van den Boom et al., 2003; Singhal and Markus, 2005). Both of these studies found that baseline CBF was lower in subjects with CADASIL than in controls. As a result, van den Boom et al. (2003) suggested that flow impairment may give rise to the development of WMHs and lacunar infarcts in patients with CADASIL.

More research specifically on the contribution of VSMCs to vascular changes in CSVD and CADASIL is essential to better understand the underlying mechanisms of these disorders.

### Stroke and post-stroke dementia

Ischaemic strokes are caused by a sudden blockage of blood flow to an area of the CNS. Having a stroke doubles the risk of dementia and the prevalence of PSD has been predicted to increase in the future because of better survival after stroke and ageing of the population (Leys et al., 2005). VSMCs appear to play an important role in the early development of ischaemic stroke and PSD due to their role in stabilising BBB integrity and perfusion, and maintaining healthy vasculature (Meng et al., 2021).

Phenotypic switching of VSMCs to a synthetic state has been shown to induce BBB disruption after ischaemic stroke in mice (Meng et al., 2021). Meng et al. (2021) found that a deficiency in myosin phosphatase target subunit 1 stimulated the phenotypic switching to a synthetic, pro-inflammatory state in human brain VSMCs and after ischaemic stroke in mice. It was also found that modifications in VSMC phenotype after stroke *in vivo* can impact the incidence, severity, pattern, and outcome of ischaemic stroke (Poittevin et al., 2014).

Furthermore, stroke-induced hypoxia can lead to the overexpression of amyloid precursor protein (APP) in VSMCs, which could exacerbate or expedite the development and progression of CAA, worsen stroke outcome, and lead to PSD (Rensink et al., 2003). Notably, before the deposition of Aβ in the brain and blood vessels, mice with APP mutations show reduced hypercapnic CVR and altered neurovascular coupling (Niwa et al., 2000). Zlokovic proposed a model to explain how cerebrovascular diseases, such as stroke, can lead to AD and dementia (Zlokovic, 2008). He proposed that stroke-induced hypoxia compromises the BBB and the overexpression of APP alters the balance between Aβ production and clearance in favour of production, which induces neuroinflammation. As a result, this may lead to synaptic dysfunction, and the accelerated onset of CAA and PSD.

Atherosclerotic plaque stability, a leading cause of ischaemic stroke, also appears to be highly dependent on the VSMC phenotype, which may either undergo apoptosis or activate the production of inflammatory mediators that can trigger plaque rupture and thrombosis (Galis and Khatri, 2002; Herrington et al., 2016). In an experimental focal cerebral ischaemia model, simvastatin promoted arteriogenesis through increased Notch receptor expression in the ischaemic cerebral arteries and peri-infarct area and it was suggested that the regulation of VSMC function through Notch signalling may be a determinant of the outcome of cerebral ischaemic disease (Zacharek et al., 2009).

Further assessment of the role of VSMCs in ischaemic brain disease and PSD may offer a new avenue for understanding the development and severity of stroke, and its progression to AD and dementia.

### Moyamoya disease

Moyamoya disease is a rare cerebrovascular disease characterised by progressive stenosis or occlusion of the terminal portion of the internal carotid arteries (Kuroda and Houkin, 2008).

Vascular smooth muscle cells in patients with MMD have shown specific differentially expressed contractile protein genes such as increased expression of smooth muscle actin and myosin heavy chain, compared with healthy control volunteers (Kang H. -S. et al., 2014). Notably, these mutations are related to VSMC adhesion, cell migration, immune response, and vascular development. The increased expression of α-smooth muscle actin may be involved in the increased proliferation of VSMCs, contributing to MMD stenosis (Guo et al., 2009). Post-mortem analysis of patients with MMD has shown degeneration in the cerebral VSMCs, and migration and morphological changes of VSMCs in the thickened intima (Lin et al., 2012).

It has also been suggested that endothelial cell dysfunction contributes to the early development of MMD and that VSMCs may require additional factors to contribute to the pathophysiology of MMD. In the analysis of VSMCs from neural crest stem cells, MMD patients and healthy control subjects were observed to have similar VSMCs proliferation, migration and contractile abilities, in contrast to the endothelial cells, which displayed distinct transcriptome profiles (Tokairin et al., 2020).

Studies have shown a significant CVR impairment in patients with MMD, based on ASL MRI and PET measurements following the injection of vasodilator acetazolamide (Deckers et al., 2021; Zhao et al., 2022). These studies showed that the CBF of patients with MMD did not increase after the injection of acetazolamide, suggesting that the cerebral vessels were already at maximal vasodilation to compensate for CBF reduction. Heyn et al. (2010) also identified decreased regional CVR in patients with MMD using CO_2_ BOLD MRI. Interestingly, unilateral surgical revascularization, a widely used preventative treatment for MMD, was found to improve PET-derived CVR in MMD patients not only in the hemisphere ipsilateral to the flow restoration, but also in the non-intervened hemisphere (Sam et al., 2015; Deckers et al., 2021). It remains that more research is required to better understand how and at what stage VSMC dysfunction may contribute to MMD development and progression.

## Extrinsic influences on vascular smooth muscle cell dysfunction

Extrinsic influences such as lifestyle choices, environmental effects, and traumatic events can impact the function, migration, and proliferation of VSMCs, and are likely to be factors in the onset and progression of neurodegenerative disorders. Many of these have been identified as risk factors for cerebrovascular and cardiovascular disorders as well as neurodegenerative disorders (Livingston et al., 2020). Understanding how extrinsic factors may influence VSMCs may inform novel interventions and therapies for preventing and arresting neurodegeneration.

### Toxins

Toxins such as those introduced to the body through cigarette smoking, alcohol or drug consumption, or air pollution are major risk factors for the early onset of neurodegenerative disorders (Mash et al., 2003; Cataldo et al., 2010; Sabia et al., 2018; Jankowska-Kieltyka et al., 2021). Exposure to toxins can also lead to profound remodelling and apoptosis of cerebral VSMCs.

Exposing cultured cerebral VSMCs to cigarette smoke extract was found to induce phenotypic switching to a pro-inflammatory, synthetic state, and suggested an avenue for the major risk that cigarette smoking causes for cerebral vascular injury such as atherosclerosis and stroke (Starke et al., 2013). In a preclinical study, nicotine exposure has been shown to stimulate abnormal growth of VSMCs and fibroblasts due to an enhanced Ang II type 1 receptor-mediated functional response (Li J. -M. et al., 2004). The same study showed that nicotine can also cause phenotypic changes of contractile VSMCs to synthetic VSMCs. The effects of nicotine on VSMCs in humans is not yet conclusive, but it has been proposed that nicotine can induce imbalances in RAS, which can cause VSMC dysfunction (Oakes et al., 2018).

High concentrations of ethanol have been shown to increase neuronal apoptosis due to increased ROS and induced apoptosis of cerebral VSMCs (Li W. et al., 2004). Similarly, exposure to even small doses of cocaine induces rapid apoptosis in cerebral VSMCs, contributing to the development of stroke and neurodegeneration (Su et al., 2003). Exposure to smaller concentrations of ethanol has been found to inhibit Notch signalling and, therefore, decrease proliferation and migration in VSMCs *in vitro* and during vessel remodelling in response to flow reduction *in vivo* in mice (Hendrickson et al., 1998; Morrow et al., 2010). In these studies, VSMC proliferation was found to increase when Notch pathways were artificially turned back on. Morrow et al. (2010) suggest that the inhibitory effect of moderate alcohol on VSMC growth may prevent vessel thickening associated with atherosclerosis and stroke. However, recent studies suggest that no level of alcohol consumption is safe in humans. These studies show that even moderate alcohol consumption has been associated with ROS generation, hypertension, stroke, vascular alterations in the peripheral and central nervous systems, cognitive decline, and more (Gill et al., 1986; Roerecke et al., 2017; Cho et al., 2022; Ohlrogge et al., 2022; Topiwala et al., 2022).

Exposure to air pollutants and ambient particulate matter has been shown to induce the transitioning of VSMCs from contractile to synthetic phenotypes, increasing cell migration and expression of proinflammatory cytokines (Ho et al., 2021). Furthermore, organic matter from ambient particulates and polycyclic aromatic hydrocarbons induce VSMC migration through ROS generation leading to vascular pathology (Ju et al., 2022). NO is a common free radical which is both present in the environment and can be produced in the body as a vital signalling molecule. In the blood vessels, NO decreases the intracellular concentration of Ca^2+^ and causes VSMC relaxation, and is thus a potent vasodilator (Zhao et al., 2015). In the environment, NO can react with other gases in the atmosphere to form nitrogen dioxide (NO2) and nitrous oxide (N2O). Notably, evidence is emerging that air pollution, including high ambient particulate matter, NO, and carbon monoxide, is also associated with increased risk of dementia and vascular damage (Cerza et al., 2019; Peters et al., 2019). More research on the effect of poor air quality on cerebral VSMCs and in neurodegenerative pathology is required to better understand the neurotoxic effect of air pollution.

### Pulsatility

Pulsatility is generated by oscillations in arterial blood flow and pressure that occur with each heartbeat. Normal haemodynamic pulsatility can be affected by age, hypertension, diabetes mellitus, hyperlipidaemia, and haematocrit levels (Jeong et al., 2018). Pulsatility factors such as blood flow-induced vessel wall shear stress, cyclic strain, and hydrostatic pressure can affect the function and phenotype of VSMCs, which has been attributed to vascular disorders (Shi and Tarbell, 2011). A recent review by Thorin-Trescases et al. (2018) suggests that age-related increased arterial stiffness may promote high pulse pressure in cerebrovasculature that leads to functional and structural alterations that could promote neuronal dysfunction and cognitive decline. Notably, a chronic increase in pulse pressure has been associated with cognitive decline and dementia (Scuteri et al., 2007; Pase et al., 2016).

Vascular smooth muscle cells have multiple sensing mechanisms to perceive mechanical stimuli generated from pulsatile stretch and transduce signals resulting in the modulation of gene expression, and cellular functions such as proliferation, apoptosis, migration, and remodelling (Haga et al., 2007; Qiu et al., 2014). A recent review found that high pulsatile shear stress induces VSMC apoptosis due to the increased release of NO from endothelial cells and low shear stress increases VSMC proliferation and migration through endothelial-released platelet-derived growth factor (Qiu et al., 2014). Other studies have shown that both the pressure amplitude and frequency of pulsatile flow can affect VSMC morphology and alterations in the cyclic oscillations modulated VSMC function and tissue stiffness (Neutel et al., 2021; Meki et al., 2022).

While more research is needed to understand the vascular changes that occur in response to pulsatile load, the effects of pulsatility on blood vessel contractility and VSMC function are notable and warrant further investigation in healthy and pathological states.

### Traumatic brain injury

Traumatic brain injury (TBI) leads to an increased risk of progressive neurodegeneration and dementia (Graham and Sharp, 2019). While the mechanisms for this are not yet clearly understood, it has been shown that TBI results in a loss in cerebrovascular tone and changes in the function of VSMCs and the neurovascular unit, which may contribute to the increased risk of neurodegenerative disease.

Traumatic brain injury has been reported to increase concentrations of endothelial-released NO, severely blunting pressure-induced vasoconstriction (Villalba et al., 2014). This was found in conjunction with a decrease in VSMC Ca^2+^ levels, which collectively led to VSMC apoptosis and profound cerebral artery dilation after TBI.

In preclinical studies, it has been demonstrated that TBI reduces gap junction coupling in VSMCs through mechanisms related to increased ROS (Yu et al., 2014). Substantial increases in hydrogen peroxide were found in VSMCs of cerebral arteries after TBI leading to decreased pressure-induced constriction of cerebral arteries in rats (Szarka et al., 2018). The impaired VSMC contractility was restored after the VSMCs were treated with a mitochondria-targeted antioxidant that blocked specific ion signalling channels in VSMCs.

A study of patients with blast-induced TBI found an overexpression of mRNA markers in contractile VSMCs and suggested that TBI leads to phenotype switching mechanisms in VSMCs of cerebral arteries, as well as prolonged hypercontraction and vasospasm (Alford et al., 2011). Alford et al. (2011) proposed that trauma causing small perturbations in tissue tension results in contractile-dominant VSMC phenotypes, while larger perturbations shift VSMCs toward a synthetic phenotype, causing large-scale remodelling.

## Therapeutic opportunities

The key to developing novel therapeutic treatments for neurodegenerative disorders depends on the understanding and investigation of the underlying molecular mechanisms. Some of these are genetic, such as in HD, DS, and CADASIL, however others such as AD, PD, ALS, and MS, can occur sporadically, and their mechanisms have been harder to establish.

Despite their variability in symptoms and progression, VSMC dysfunction appears to be a common link between these disorders and could prove to be a promising target for intervention. Studies have shown that targeting the vascular tissue with drugs may be effective at modulating and/or preventing neurodegenerative disorders such as AD and PD (Speer and Ratan, 2009; Aguilar-Pineda et al., 2021a). It has also been suggested that intervening during the differentiation, proliferation, and/or migration mechanisms of VSMCs could be a therapeutic strategy to reduce pathological VSMC functions while maintaining their stabilising roles in CBF (Worssam and Jørgensen, 2021). Here, we will review recent insights on VSMC-based therapeutics in vascular pathology and discuss how VSMCs could be targeted to intervene in the development and progression of neurodegenerative disorders.

### Signalling pathways

It has been proposed that targeting VSMC signalling mechanisms could be protective against the onset of neurodegeneration (Marsboom and Archer, 2008; Lin et al., 2021). Notably, increased VSMC proliferation and migration exhibited in AD, ALS, and MMD can be harmful to cerebrovascular function and may contribute to the accumulation of toxic plaques in the central nervous system (Krettek et al., 1997; Guo et al., 2009; Aguilar-Pineda et al., 2021b). In other pathologies, such as CADASIL, a dramatic reduction in VSMC proliferation can lead to vessel thinning and reduced CVR amplitude (Liem et al., 2009; Panahi et al., 2018). VSMCs have several signalling pathways to control cell function and proliferation, however a few players have been identified as key contributors in VSMC proliferation.

Vascular smooth muscle cell proliferation and migration can be manipulated by inhibiting the degradation of cyclin-dependent kinase (CDK) with pharmaceuticals like thiazolidinediones, which have been developed for treating type II diabetes (Wakino et al., 2000). These drugs are also peroxisome proliferator-activated receptor γ (PPARγ) agonists and the activation of PPARγ in VSMCs has been shown to inhibit synthetic VSMC proliferation and migration, which may have vascular-protective effects (Law et al., 2000; Yang et al., 2013). The CDK pathway is also involved in telomere activity, which stabilises chromosomes. Telomerase reverse transcriptase (TERT) phosphorylation, which is upregulated during and following hypoxia, has been shown to increase VSMC proliferation (Minamino et al., 2001). Some evidence has shown that inhibiting TERT *in vivo*, such as via the CDK pathway, may reduce vascular disease (Fuster and Andrés, 2006).

The transient receptor potential vanilloid (TRPV) family is expressed abundantly in cerebral artery VSMCs and has been shown to be an important mediator of vascular tone (Guibert et al., 2011). The overexpression of TRPV1 has been found to inhibit Ang II induced VSMC migration and phenotype transitions from a contractile to secretory state (Wang and Jia, 2020). Another study found that activated TRPV1 inhibited VSMC proliferation, migration, and ROS production by upregulating the expression of PPARα (Zhou et al., 2021). Additionally, TRPV4 channel activity has been found to increase vasoconstriction and elevate resting blood pressure in mice and patients with hypertension (Chen et al., 2022).

RhoA/Rho-kinase have been shown to modulate VSMC phenotype switching including affecting proliferation, dedifferentiation, apoptosis, and migration in VSMCs, which is discussed extensively in the review paper by Sawma et al. (2022). Notably, the inhibition of Rho signalling using fasudil hydrochloride is currently used clinically for the treatment of vasospasm (Montagnoli et al., 2021). It has been suggested that it may also reduce inflammation and lesion volume in MS (Aktas et al., 2010).

Interestingly, berberine has been increasingly found to have vascular- and neuro-protective effects against neurodegenerative pathology, which could be attributed to its effects on signalling pathways (Kumar et al., 2016; Ye C. et al., 2021). Berberine is an organic compound in classical natural medicine, proven by modern research to have various pharmacological effects, including antiviral, anti-inflammatory, and lipid regulation (Ye Y. et al., 2021). Preclinically, berberine was found to affect smooth muscle contractility by regulating the VSMC handling of intracellular Ca^2+^ (Ma et al., 2016). *In vitro*, berberine inhibited early signalling pathways induced by VSMC injury, reducing the contraction and proliferation of dysfunctional VSMCs (Liang et al., 2006). Other studies have shown that berberine inhibits induced VSMC proliferation via the PPARγ signalling pathway by stimulating a beneficial increase in NO (Calvier et al., 2017; Qiu et al., 2017).

Notch signalling has gained significant attention in the context of vascular biology and has been identified as a possible culprit for VSMC and pericyte reduction seen in some neurodegenerative disorders (Lin et al., 2021). As mentioned earlier, Notch3 is a gene involved in the pathogenesis of CADASIL, though it is currently not known if the mutations in Notch3 are causative (Ihalainen et al., 2007). One study showed that Notch3 is necessary for pericyte proliferation in zebrafish (Wang et al., 2014). In contrast, another study found that while Notch3 mutations disrupt and compromise the BBB, the mutations have no direct effect on mural cells (Henshall et al., 2015). While very minimal research has been conducted on Notch-based therapeutics in neurodegeneration, targeting Notch receptors in mice models of inflammatory disorders that share features with human MS has shown very minimal side effects while safely preventing and reversing inflammation (Minter et al., 2005; Sandy et al., 2013).

Renin-angiotensin system may also be a potential target for therapeutic intervention for VSMC dysfunction. Angiotensin-converting enzyme inhibitors and angiotensin receptor blockers have been shown as effective modulators of AD in preclinical models (Lee et al., 2020). In a mouse model, angiotensin receptor blockers alone were not found to restore Aβ-related cognitive and cerebrovascular deficits (Royea et al., 2020). However, captopril (another angiotensin converting enzyme inhibitor) alone was found to reduce Aβ deposition *in vitro* and *in vivo*, and improve cognition in animal models (AbdAlla et al., 2013; Asraf et al., 2018).

Another major signalling molecule, adenosine, is synthesised by VSMCs and endothelial cells via multiple pathways, and may be useful for controlling VSMC growth in vascular disease (Pearson et al., 1980). Studies have shown that adenosine inhibits VSMC growth and that a reduction in local adenosine levels may initiate VSMC growth and contribute to the vascular remodelling in hypertension and atherosclerosis (Dubey et al., 1996). Additionally, abnormal adenosine homeostasis has been observed in PD, HD, and ALS (Yoshida et al., 1999; Armentero et al., 2011; Lee and Chern, 2014). The review by Chang et al. (2021) summarises this topic more extensively and highlights the therapeutic opportunities for targeting adenosine homeostasis in neurodegenerative disorders.

Vascular smooth muscle cells are crucial in hypertension progression and the response of VSMCs to statins, most commonly used to treat and prevent hypertension, has therefore been more extensively researched, though only more recently in the context of neurodegeneration (Sarafidis et al., 2007). Statins can affect several components of RAS pathways in conjunction with lowering low-density lipoprotein cholesterol. Notably, statins can inhibit the harmful overexpression of Ang II and AT1 receptors, and support healthy VSMC contraction (Kiaie et al., 2021). It has been shown that statins can inhibit VSMC proliferation and reduce vascular inflammation, likely by decreasing the sensitivity of VSMCs to Ca^2+^ leading to structural remodelling, and reduced vascular constriction (Garg and Hassid, 1989; Kang S. et al., 2014). As a result, statins may have vasodilatory and antithrombotic properties, which could enhance collateral circulation and improve CVR, notably in cases where VSMCs are hypercontractile (Giannopoulos et al., 2012). It appears that statins may also reduce the accumulation of Aβ in the brain and have protective effects against cognitive decline, however it is unclear which mechanisms drive these outcomes (Schultz et al., 2018).

Here we have identified signalling pathways such as PPARγ, CDK, Notch, Rho, TRPV, adenosine, and RAS, which may be potential targets for intervening in VSMC dysfunction. Further research is critical to clearly understand the effect of these signalling pathways on VSMC physiology in both healthy and diseased states.

### Inhibiting reactive oxygen species

Another method of inhibiting VSMC phenotype transitions could be through the inhibition of ROS. In VSMCs, ROS are known to mediate many physiological processes such as phenotypic switching and signalling pathways, as well as pathophysiological processes, including migration, proliferation, apoptosis, and inflammatory cytokine secretion (Rao and Berk, 1992; Tsai et al., 1996; Griendling et al., 1997).

In mice, the inhibition of ROS via the oral administration of the antioxidant drug apocynin suppresses proinflammatory and proliferative synthetic VSMCs associated with vascular disorders (Pi et al., 2013). While the precise mechanisms underlying the effect of ROS remain unclear, the same study suggested that ROS induces VSMC phenotype switching through the upregulation of toll-like receptor 4 (TLR4) in endothelial cells, which can be modulated using drugs such as apocynin. Shimokawa (2013) showed that the growth factor cyclophilin A contributes to ROS effects in VSMC by promoting growth and, as a result, the effect of ROS on VSMC function may be possible by blocking the cyclophilin A signalling or binding pathways.

It has been demonstrated that ROS mediates vascular contraction and remodelling by activating Rho-signalling (Jin et al., 2004). For example, the long-term blockage of the Rho-kinase pathway was shown to inhibit induced vascular remodelling in mice by blocking the synthesis of ROS, such as NO (Ikegaki et al., 2001). As a result, the Rho pathways may be a target for inhibiting ROS.

These findings suggest that novel pharmacological strategies aimed at mediating the effect of VSMC phenotype switching through the inhibition of ROS may have therapeutic potentials in treating VSMC dysfunction.

### Gene therapy

Gene therapy is an emerging therapeutic tool by which functional genetic material is delivered to specific cells to correct a defective gene. Several clinical studies have tested gene therapy for treating neurodegenerative disorders, including PD and AD, though the efficacy of these approaches has yet to be determined (Bartus et al., 2013; Palfi et al., 2014; Tuszynski et al., 2015).

Among the most promising of potential gene therapy targets for VSMC dysfunction is SERCA2a, the SERCA isoform present in the nervous system, which has been approved as a human gene therapy target to treat heart failure (Jessup et al., 2011). This has been attributed to the normalisation of Ca^2+^ cycling in VSMCs and inhibiting Ca^2+^-dependent transcription factors (Merlet et al., 2013). This could be used to target VSMC dysfunction as forced expression of SERCA2a in contractile VSMCs has been shown to prevent injury-induced dedifferentiation toward a synthetic, inflammatory phenotype, whereas forced expression in synthetic VSMCs reduces their proliferation and migration, but has no effect on their phenotype (Bobe et al., 2011).

The review papers by Piguet et al. (2017), Martier and Konstantinova (2020) explore the past, current, and future perspectives of gene therapy for neurodegenerative disorders. Importantly, it has been noted that delivering therapies administered before the onset of neurodegeneration is essential to improve the efficacy of gene therapies (Martier and Konstantinova, 2020). Therefore, the discovery of biomarkers for early detection of neurodegenerative disorders and a better understanding of factors leading to these disorders is essential to improve clinical applications of gene therapy.

## Concluding remarks

In this work, we reviewed the role of VSMCs in the most common neurodegenerative disorders and identified mechanisms by which VSMC dysfunction may contribute to neurodegeneration. In particular, changes in VSMC phenotype, signalling pathways, and interactions with ROS are seen in a host of neurodegenerative disorders and could serve as targets for novel therapeutic interventions. We identified CVR and CBF measurements as promising techniques by which VSMC dysfunction could be characterised *in vivo*, but further research is required to understand how to isolate VSMC contributions. Note that correlations between VSMC dysfunction and some of the disorders were more extensively studied than others, and methodologies varied greatly between them. Furthermore, inferences drawn from some of the studies analysed in this review should be taken with caution as not all of them were specifically assessing VSMC dysfunction in this context, and it remains unclear whether VSMC dysfunction is a cause or a consequence of these disorders. In conclusion, the evidence collected here highlights the critical role of VSMC dysfunction in the development and progression of neurodegeneration, and the need for more investigation into VSMCs and cerebrovascular dynamics in neurodegenerative disease.

## Author contributions

GH, JP, and DB contributed to the conception and initial design of the manuscript. GH performed the search and selection of the relevant studies and wrote the manuscript. JP, SNS, CW, and SS contributed to the manuscript revision and editing. All authors read and approved the submitted manuscript.

## Abbreviations

VSMC: vascular smooth muscle cell
CBF: cerebral blood flow
BBB: blood brain barrier
CVR: cerebrovascular reactivity
MRI: magnetic resonance imaging
BOLD: blood-oxygen-level-dependent
ASL: arterial spin labelling
TCD: transcranial doppler
ROS: reactive oxygen species
Ang II: angiotensin II
A β: amyloid-beta
AT1: type-1 Ang II receptor
APP: amyloid precursor protein
AD: Alzheimer’s disease
APOE: apolipoprotein E
CAA: cerebral amyloid angiopathy
ALS: amyotrophic lateral sclerosis
MS: multiple sclerosis
HD: Huntington’s disease
CSVD: cerebral small vessel disease
PSD: post-stroke dementia
DS: Down’s syndrome
CADASIL: cerebral autosomal dominant arteriopathy with subcortical infarcts and leukoencephalopathy
LBD: lewy body dementia
MMD: Moyamoya disease
WMH: white matter hyperintensities
SERCA2: sarco-endoplasmic reticulum calcium ATPase isoform 2
PPAR γ: peroxisome proliferator-activated receptor γ
CDK: cyclin-dependent kinase
TERT: telomerase reverse transcriptase.
